# Immune Thrombocytopenia Associated with Hepatitis B Virus and Autoimmune Hepatitis and Recovery of Platelet Count following Liver Transplantation

**DOI:** 10.1155/2021/8484106

**Published:** 2021-09-15

**Authors:** Chloe Nobuhara, Diana M. Cardona, Murat O. Arcasoy, Carl L. Berg, Andrew S. Barbas

**Affiliations:** ^1^Department of Surgery, Duke University Medical Center, Durham, NC, USA; ^2^Department of Pathology, Duke University Medical Center, Durham, NC, USA; ^3^Department of Medicine, Duke University Medical Center, Durham, NC, USA

## Abstract

Immune thrombocytopenia is a consumptive coagulopathy that can be either idiopathic or associated with infectious or autoimmune etiologies. Here, we present a case of immune thrombocytopenia in the setting of acute liver failure due to coexisting diagnoses of hepatitis B virus and autoimmune hepatitis. Our patient underwent orthotopic liver transplantation and recovered hemostatic platelet counts after treatment with romiplostim, a thrombopoietin receptor agonist, 51 days after transplantation. To our knowledge, this is the first case report of immune thrombocytopenia secondary to both hepatitis B virus and autoimmune hepatitis in a patient with acute liver failure.

## 1. Introduction

Patients with acute liver failure may present with thrombocytopenia for a variety of reasons: decreased thrombopoietin (TPO) production, splenic sequestration, or, more rarely, consumptive coagulopathy. Immune thrombocytopenia (ITP) is an autoimmune coagulopathy mediated by platelet autoantibodies that both destroy platelets and inhibit their formation [1]. While most cases (approximately 80%) are idiopathic, ITP can also be secondary to various infectious and autoimmune conditions [2]. Both hepatitis B virus (HBV) and autoimmune hepatitis (AIH) have independently been reported as causes of secondary ITP in the literature, albeit independently [3–5].

Here, we report a novel case of the onset of ITP in a patient with acute liver failure who had positive HBV serologic markers as well as AIH on explanted liver histology. This patient presented with acute liver failure and severe thrombocytopenia and underwent orthotopic liver transplantation (OLT) under two days after admission to our institution. She received multiple units of platelets under a massive transfusion protocol which were subsequently destroyed by presumed ITP. Treatment was refractory to prednisone and intravenous immunoglobulin (IVIG), and she was initiated on romiplostim, a TPO receptor agonist. She achieved platelet counts of >100 × 10^9^/L on postoperative day (POD) 51. This case highlights the importance of determining the etiology of thrombocytopenia in patients with acute liver failure and reports the potential coexistence of HBV and AIH as secondary causes of immune thrombocytopenia.

## 2. Methods and Materials

This case report is a single, deidentified patient case. It is therefore exempt from approval from our Institutional Review Board.

## 3. Case Report

A 48-year-old woman with no prior liver disease presented to an outside hospital with a two-week history of worsening fatigue, jaundice, and heavy menstrual bleeding. Laboratory values were significant for severe thrombocytopenia and coagulopathy, and she was found to have transaminitis and positive HBV markers on serology. She was initiated on N-acetylcysteine and vitamin K before being transferred to our institution for emergent liver transplant evaluation.

Upon arrival to our institution, she was afebrile and normotensive with oxygen saturation 90% percent on room air. Laboratory findings were remarkable on admission for the following: hemoglobin, 9.3; platelets, 10; PT‐INR > 10; D-dimer, 55,418; aspartate aminotransferase (AST), 3,061; alanine aminotransferase (ALT); 1,519; antismooth muscle antibody (SMA) positive at a 1 : 60 dilution; antinuclear antibodies (ANA) negative; HBV core antibody positive; surface antigen positive; and HBV DNA detectable at 1,010 units (Table 1). Her physical examination was notable for jaundice and asterixis. She subsequently required intubation for airway protection in the setting of hepatic encephalopathy and obtundation.

Her past medical history was significant for a pulmonary embolism of unknown etiology treated with rivaroxaban, which was discontinued a month prior. She denied alcohol use, intravenous drug use, prior blood transfusions, or unprotected sexual contact with new partners. She did not know if she had been previously vaccinated against HBV and was never told that she had HBV. Her husband was born in Africa, and it was possible that he acquired it at birth, but she was separated from her husband at the time. She had no known history of genetic liver disease or family history of autoimmune disease.

The patient underwent orthotopic liver transplantation two days after admission. Her calculated MELD score at the time was 40. She received a donation after brainstem death whole liver with standard arterial anatomy, duct-to-duct biliary anastomosis. She received hepatitis B virus immunoglobulin 9360 IU during the anhepatic phase intraoperatively. Estimated blood loss was 2000 mL, and massive transfusion protocol was initiated with broad-spectrum antibiotic coverage. The rest of the transplant was uncomplicated.

**Figure 1:**
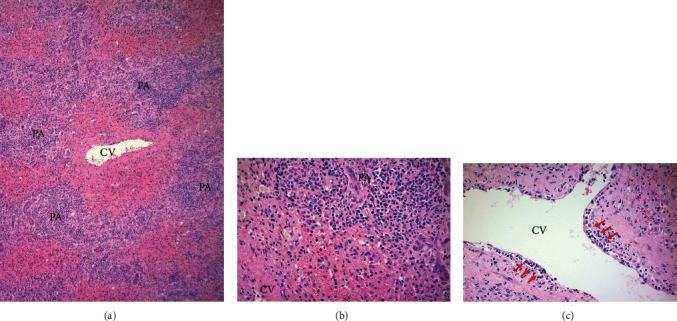
(a) The liver reveals massive hepatocyte necrosis. The lobular parenchyma surrounding the central vein (CV) is devoid of hepatocytes, and the portal areas (PA) have collapsed centrally. The portal areas are involved by a dense chronic inflammatory infiltrate and bile ductular proliferation (H&E; 40x). (b) Magnified view of a portal area (PA) and limiting plate with the lobular necrosis and portion of the CV. There are numerous plasma cells with interface activity (H&E; 200x). (c) A central vein is involved by endothelialitis, depicted by chains of lymphocytes (arrows) undermining the endothelium (H&E; 200x).

Based on the criteria before histologic evaluation, this patient's score on the Autoimmune Hepatitis Scoring System was 7 (female +2, ALP/ALT ratio +2, IgG level +0, smooth muscle antibody +3, antimitochondrial antibody +0, viral markers -3, drugs +1, alcohol +2, immune disease +0), consistent with possible AIH. Histologic evaluation of the explanted liver was compatible with autoimmune hepatitis (Figure 1). There was massive hepatocyte necrosis involving over 85% of the parenchyma. Inflammation was predominantly lymphoplasmacytic with central venous endothelialitis, compatible with AIH. Notably, there was no definitive morphologic evidence of HBV. There were no ground glass inclusions or other viral cytopathic effect, and immunohistochemistry revealed that the liver was negative for HBV surface antigen.

**Figure 2:**
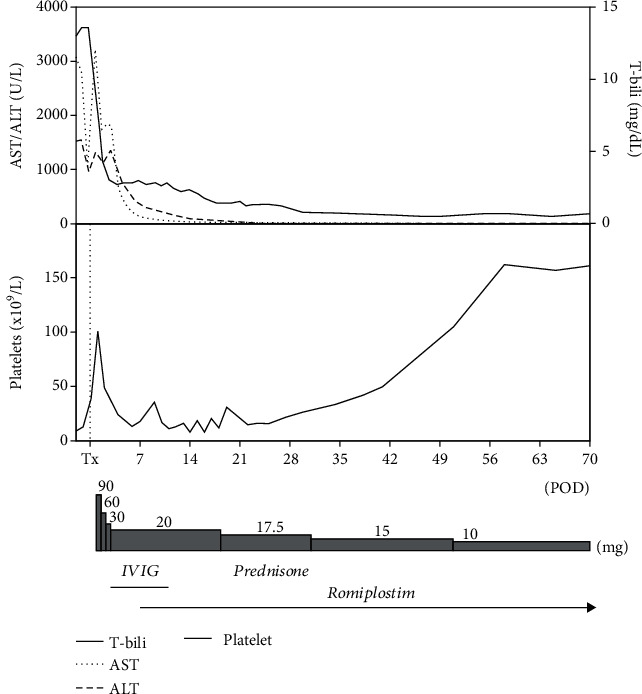
Selected laboratory values by postoperative day (POD). Tx: liver transplant; AST: aspartate aminotransferase; ALT: alanine aminotransferase; T-Bili: total bilirubin; IVIG: intravenous immunoglobulin.

Her postoperative course was complicated by severe thrombocytopenia and anemia in the setting of vaginal bleeding. Hematology had a high clinical suspicion for ITP. However, the risk of bleeding during a bone marrow biopsy was deemed too high to perform the procedure for diagnosis alone. She was already on prednisone for transplant immunosuppression, which is first-line therapy for ITP [6]. Treatment was initiated with intravenous IVIG, and she was given six courses. Romiplostim 500 mcg injection was initiated on POD 9 without adverse effects. Her platelet counts recovered from 10 × 10^9^/L (POD -2) to 106 × 10^9^/L (POD 51) as shown in Figure 2. Notably, there was a transient increase to platelets of 101 × 10^9^/L on POD 1 following massive transfusion protocol initiated during liver transplantation, and the subsequent antibody mediated consumption of these platelets to 48 × 10^9^/L on POD 2.

## 4. Discussion

Patients with acute liver failure often present with thrombocytopenia. This case represents a unique scenario of ITP associated with coexisting diagnoses of HBV and AIH, highlighting the importance of determining the etiology of thrombocytopenia in a patient with acute liver failure.

The diagnosis of ITP is clinical; it is defined by a peripheral blood platelet count of <100 × 10^9^/L after excluding other causes of thrombocytopenia [1]. The development of antiplatelet antibodies in patients with ITP is often seen in the setting of infection (e.g., via molecular mimicry) or other autoimmune conditions [2]. However, to our knowledge, this is the first report of ITP associated with both AIH and HBV diagnoses. AIH has been reported to present in patients with chronic hepatitis B and C virus [7]. HBV and HCV can lead to the development of non-organ-specific autoantibodies (NOSA), particularly ANA and SMA [8]. In some cases, this may be attributed to overdiagnosis of AIH in patients with viral hepatitis based on serology alone. However, we believe that our patient was a true case of AIH since she had anti-SMA antibodies at a 1 : 160 titer plus confirmatory explant histology.

In adults with newly diagnosed ITP and a platelet count of <30 × 10^9^/L (our patient presented platelets of 10 × 10^9^/L), the 2019 American Society of Hematology guidelines recommend corticosteroids with IVIG when a rapid increase in platelet count is required [6]. Our patient was given a prednisone induction of 90 mg and gradually tapered until discontinuation on POD 128 as standard practice for liver transplantation candidates (Figure 2). IVIG was initiated at a dose of 1 g/kg on POD 6 with repeated doses until POD 12 with minimal rise in platelet counts (13 to 13 × 10^9^/L).

Because her platelet counts were refractory to first-line treatment, she was initiated on romiplostim on POD 9. Historically, second-line therapy for ITP has involved splenectomy which removes the source of antiplatelet antibody generation and prevents platelet sequestration. The utilization of splenectomy for refractory ITP has decreased over the past few decades due to the upfront risks of surgical morbidity and the rise of newer medical treatments. In 2019, the US Food and Drug Administration (FDA) expanded the use criteria for romiplostim, a TPO receptor agonist, to adults with refractory ITP [6]. Romiplostim is a peptide-antibody fusion product that is administered via intravenous infusion and is reported to be well-tolerated with high patient satisfaction and quality of life [9].

We present a novel case of the onset of ITP in a patient with acute liver failure who had positive HBV serologic markers as well as AIH on explanted liver histology and the subsequent resolution to hemostatic platelet counts after liver transplantation. This report outlines two distinct learning points. First, the possibility of ITP presenting mechanistically secondary to both HBV and AIH, which has not previously been reported in the literature. Many patients in acute liver failure also present with thrombocytopenia; thus, the possibility of this unique etiology should be reported. Second, in our patient with ITP secondary to HBV and AIH, liver transplantation was safe and platelet counts recovered after combination with romiplostim. In such patients, recovery of platelet counts following liver transplantation is feasible and may address the underlying cause of ITP.

## Figures and Tables

**Table 1:** Serologic markers on admission.

*Hematology*			
White blood cell	5.9		×10^9^/L
Red blood cell	3.46	(L)	×10^12^/L
Hemoglobin	9.3	(L)	g/dL
Hematocrit	25.7	(L)	%
Platelets	10	(LL)	×10^9^/L

*Coagulation*			
PT	>120	(H)	sec
PT-INR	>10	(HH)	
aPTT	43.8	(H)	sec
Fibrinogen	159	(L)	mg/dL
D-dimer	55,418	(H)	FEU

*Biochemistry*			
Albumin	2.1	(L)	g/dL
Bilirubin, total	12.9	(H)	mg/dL
Bilirubin, conjugated	4.7	(H)	mg/dL
AST	3,061	(H)	U/L
ALT	1,519	(H)	U/L
ALP	115	(H)	U/L
BUN	<1	(L)	mg/dL
Creatinine	0.6		mg/dL

*Immunology*			
ANA	Negative		
Anti-SM Ab	Positive		
Anti-SM Ab titer	1 : 160		
Antimitochondrial	Negative		
IgG	1,280		mg/dL
IgM	97		mg/dL
IgG4	108		mg/dL

*Infectious*			
HBV core Ab	(+)		
HBV surface Ab	(-)		
HBV surface Ag	(+)		
HBV DNA	1,010		IU/mL
HCV RNA	(-)		
HIV Ab/Ag	(-)		
CMV IgG	(+)		
EBV IgG	(+)		

PT: prothrombin time; INR: international normalized ratio; aPTT: activated partial thromboplastin time; AST: aspartate aminotransferase; ALT: alanine aminotransferase; SM: antismooth muscle; HBV: hepatitis B virus; HCV: hepatitis C virus; CMV: cytomegalovirus; EBV: Epstein-Barr virus.

## Data Availability

No data were used to support this study.

## Abbreviations

AIH: Autoimmune hepatitis
ALT: Alanine aminotransferase
ANA: Antinuclear antibodies
AST: Aspartate aminotransferase
HBV: Hepatitis B virus
ITP: Immune thrombocytopenia
IVIG: Immunoglobulin
MELD: Model for end-stage liver disease
NOSA: Non-organ-specific autoantibodies
POD: Postoperative day
SMA: Antismooth muscle
TPO: Thrombopoietin.
